# Supplementary material for: Meta-Analysis and Gene Set Enrichment Relative to ER Status Reveal Elevated Activity of MYC and E2F in the “Basal” Breast Cancer Subgroup
Source: PLoS One. 2009 Mar 9;4(3):e4710. doi: 10.1371/journal.pone.0004710 (PMC2650420; doi:10.1371/journal.pone.0004710)
Supplement: Dataset S1 — GSEA results from first screen. The file contains a zipped archive of the HTML results of the second GSEA screen. Reports for enriched or depleted datasets may be accessed from the “index.html” link within the my_analysis.GseaPreranked.1219975950625.rpt.zip ZIP archive. Enrichment plots are available for each of the 50 most enriched and depleted gene sets. (13.32 MB ZIP) [file pone.0004710.s001.zip › my_analysis.GseaPreranked.1219975950625.rpt.zip!//GENOTOXINS_ALL_4HRS_REG.html]

Details for gene set GENOTOXINS\_ALL\_4HRS\_REG[GSEA]

|  || Dataset | zSco.ERneg.to.ERpos\_collapsed |
| Phenotype | NoPhenotypeAvailable |
| Upregulated in class | na\_pos |
| GeneSet | GENOTOXINS\_ALL\_4HRS\_REG |
| Enrichment Score (ES) | 0.74053586 |
| Normalized Enrichment Score (NES) | 2.230998 |
| Nominal p-value | 0.0 |
| FDR q-value | 0.0 |
| FWER p-Value | 0.0 |
Table: GSEA Results Summary

  

Fig 1: Enrichment plot: GENOTOXINS\_ALL\_4HRS\_REG      
 Profile of the Running ES Score & Positions of GeneSet Members on the Rank Ordered List

  

| PROBE | GENE SYMBOL | GENE\_TITLE | RANK IN GENE LIST | RANK METRIC SCORE | RUNNING ES | CORE ENRICHMENT || 1 | CDC20 |  |  | 9 | 7.151 | 0.1017 | Yes |
| 2 | BUB1 |  |  | 41 | 6.445 | 0.1916 | Yes |
| 3 | MTHFD2 |  |  | 205 | 5.307 | 0.2554 | Yes |
| 4 | PTTG1 |  |  | 225 | 5.206 | 0.3284 | Yes |
| 5 | CKS2 |  |  | 239 | 5.145 | 0.4011 | Yes |
| 6 | IFNAR2 |  |  | 325 | 4.864 | 0.4644 | Yes |
| 7 | SYNCRIP |  |  | 402 | 4.679 | 0.5256 | Yes |
| 8 | AURKA |  |  | 460 | 4.483 | 0.5855 | Yes |
| 9 | PRC1 |  |  | 543 | 4.253 | 0.6403 | Yes |
| 10 | CUL1 |  |  | 803 | 3.773 | 0.6748 | Yes |
| 11 | SMG5 |  |  | 1416 | 3.027 | 0.6721 | Yes |
| 12 | NEK2 |  |  | 1703 | 2.758 | 0.6901 | Yes |
| 13 | CDC25C |  |  | 1794 | 2.700 | 0.7220 | Yes |
| 14 | ECT2 |  |  | 2026 | 2.509 | 0.7405 | Yes |
| 15 | CDT1 |  |  | 2522 | 2.190 | 0.7347 | No |
| 16 | ITGB7 |  |  | 3076 | 1.896 | 0.7202 | No |
| 17 | MAPRE1 |  |  | 5601 | 0.944 | 0.5439 | No |
| 18 | TNNC1 |  |  | 7187 | 0.422 | 0.4308 | No |
| 19 | ALAS1 |  |  | 7540 | 0.292 | 0.4085 | No |
| 20 | KIF22 |  |  | 8725 | -0.180 | 0.3221 | No |
| 21 | HMGN1 |  |  | 9754 | -0.697 | 0.2547 | No |
| 22 | TRAFD1 |  |  | 10151 | -0.930 | 0.2383 | No |
Table: GSEA details [plain text format]

  

Fig 2: GENOTOXINS\_ALL\_4HRS\_REG: Random ES distribution      
 Gene set null distribution of ES for **GENOTOXINS\_ALL\_4HRS\_REG**
